# Advanced ultrasonography technologies to assess the effects of radiofrequency ablation on hepatocellular carcinoma

**DOI:** 10.2478/raon-2013-0033

**Published:** 2013-07-30

**Authors:** Nobuyuki Toshikuni, Hisakazu Shiroeda, Kazuaki Ozaki, Yasuhiro Matsue, Takahiro Minato, Tomoe Nomura, Nobuhiko Hayashi, Tomiyasu Arisawa, Mikihiro Tsutsumi

**Affiliations:** Department of Gastroenterology, Kanazawa Medical University, Ishikawa, Japan

**Keywords:** hepatocellular carcinoma, radiofrequency ablation, contrast-enhanced ultrasonography, real-time virtual sonography, three-dimensional ultrasonography

## Abstract

**Background:**

Radiofrequency ablation (RFA) is a curative therapy for hepatocellular carcinoma (HCC). In RFA, ultrasonography (US) is most commonly used to guide tumor puncture, while its effects are assessed using dynamic computed tomography or magnetic resonance. The differences in modalities used for RFA and assessment of its effects complicate RFA. We developed a method for assessing the effects of RFA on HCC by combining contrast-enhanced (CE) US and real-time virtual sonography with three-dimensional US data.

**Patients and methods:**

Before RFA, we performed a sweep scan of the target HCC nodule and the surrounding hepatic parenchyma to generate three-dimensional US data. After RFA, we synchronized multi-planar reconstruction images derived from stored three-dimensional US data with real-time US images on the same US monitor and performed CEUS and real-time virtual sonography. Using a marking function, we drew a sphere marker along the target HCC nodule contour on pre-treatment US- multi-planar reconstruction images so that the automatically synchronized sphere marker represented the original HCC nodule contour on post-treatment real-time CEUS images. Ablation was considered sufficient when an avascular area with a margin of several millimeters in all directions surrounded the sphere marker on CEUS.

**Results:**

This method was feasible and useful for assessing therapeutic effects in 13 consecutive patients with HCC who underwent RFA. In 2 patients who underwent multiple sessions of RFA, HCC-nodule portions requiring additional RFA were easily identified on US images.

**Conclusions:**

This method using advanced US technologies will facilitate assessment of the effects of RFA on HCC.

## Introduction

Percutaneous radiofrequency ablation (RFA) is a minimally invasive curative therapy for unresectable hepatocellular carcinoma (HCC).^1^ During RFA, ultrasonography (US) is most commonly used to guide tumor puncture, while side-by-side comparisons of pre- and post-treatment contrast-enhanced computed tomography (CT) or magnetic resonance (MR) images are typically used to assess the therapeutic effects of RFA. Differences in the modalities for RFA and the techniques for assessing its effects complicate RFA. Furthermore, side-by-side comparisons of images can often make assessment of ablated margins difficult.

Recent advances in imaging modalities have enabled the synthesis of high-resolution multiplanar reconstruction (MPR) images from three-dimensional (3D) data.^2^ This technology and position-tracking systems with magnetic navigation allow the synchronization of CT- or MR-MPR images with real-time US images or real-time virtual sonography (RVS) images.^3^ With this method, one can utilize MPR images during US examinations. RVS has been shown to be useful for detecting small hepatic nodules^4^ and guiding the puncture of HCC nodules that are difficult to detect with conventional US but detectable with CT and/or MR imaging.^5^–^8^

Contrast-enhanced US (CEUS) with perflubutane (Sonazoid; GE Healthcare, Oslo, Norway), a microbubble contrast agent, has been routinely used in Japan to scrutinize hepatic tumors.^9^ Recent studies have demonstrated that the use of this contrast agent during CEUS improved HCC detection by conventional US, thereby facilitating accurate nodule puncture.^10^–^12^

The combination of CEUS and RVS provides information on the accurate position and vascular flow of the target lesion. We hypothesized that CEUS and RVS using 3DUS data may be useful for assessing the effects of RFA and reducing its complexity. Here we describe our preliminary results of this promising method.

## Patients and methods

### In vitro experiments using a phantom model

To verify whether CEUS and RVS using 3DUS data could be applied to assess the effects of RFA on HCC, an operator (N.T.) with 20 years of experience in performing US examinations used 3DUS data from a phantom model (Model 057 Triple Modality 3D Abdominal Phantom; CIRS, Norfolk, USA) that contained structures representing abdominal organs to conduct an RVS synchronization experiment. The operator performed a manual sweep scan with a US machine (HI VISION Preirus; Hitachi Medical Corporation, Tokyo, Japan) and a convex probe (EUP-C715; Hitachi Medical Corporation) at an arbitrary site on the surface of the phantom model. The scan time was set for 15 s. The 3DUS data sets were automatically generated and manually stored on the hard disk of the US machine. Immediately after storage of the 3D data, the same site was scanned and examined to determine whether US-MPR images from the 3D data were synchronized with real-time US images on the same US monitor. The US machine had a marking function that automatically displayed synchronized straight and spherical markers on US-MPR and real-time US images, respectively. The size of a spherical marker was designed to change inversely with the distance between the center of the marker and scanning section. Then, the operator drew a spherical marker along the contour of a spherical structure to show its maximum diameter on the US-MPR image and determined that the synchronized spherical markers were displayed along the contour of the spherical structure in all directions on the synchronized US-MPR and real-time US images. The operator repeated the same procedures five times.

### Patients

This study was conducted in accordance with the guidelines of the Declaration of Helsinki and the standards of the institutional ethics committee. Informed consent was obtained from all patients. The study included 13 consecutive patients with 14 HCC nodules who underwent RFA between December 2011 and April 2012. HCC diagnoses were based on the results of CEUS, dynamic CT, and dynamic MR imaging.^13^ RFA was indicated according to the Clinical Practice Guidelines for Hepatocellular Carcinoma (the J-HCC guidelines), the first evidence-based clinical practice guidelines for the treatment of HCC in Japan, which were compiled by an expert panel supported by the Japanese Ministry of Health, Labour and Welfare.^14^

### RFA

Two operators (N.T. and H.S.) with 7 years and 3 years of experience with the technique performed RFA using a cooled-tip RFA system (Covidien, Mansfield, MA, USA) on the enrolled patients. A 17-gauge, internally cooled-tip electrode with a 2- or 3-cm tip that was attached to a 480-kHz monopolar RF generator was percutaneously inserted into the target HCC under real-time US guidance using an US machine (HI VISION Preirus; Hitachi Medical Corporation) and a 3.5-MHz microconvex probe (EUP-B512; Hitachi Medical Corporation). The generator output was slowly increased to 80–120 watts and maintained for up to 12 min. In some cases, multiple RF electrode insertions were required to obtain sufficient ablation.

### CEUS and RVS using 3DUS data

Before RFA, the operators (N.T. and H.S.) and their assistants (K.O. and N.H.) used a US machine (HI VISION Preirus) to perform planning B-mode US and CEUS with perflubutane (Sonazoid). A manual sweep scan with a convex probe (EUP-C715; Hitachi Medical Corporation) was performed at the planned puncture site in B-mode US and during the vascular and Kupffer phases of CEUS. The sweep scan covered the target HCC nodule and surrounding hepatic parenchyma. The scan time was set for 15 s. CEUS, RVS, and the stored 3DUS data were used by the operators (N.T. and H.S.) to assess by consensus the therapeutic effects of a single RFA session 1 to 2 days after the session. A scan with a US machine (HI VISION Preirus) and a convex probe (EUP-C715) was performed at the puncture site. We selected the data set with the clearest images of the target HCC nodule. While a patient held his/her breath, the operators manually moved and rotated the 3DUS data to synchronize the US-MPR images with real-time US images on the same US monitor. Straight markers were used to adjust both-side images; the markers were drawn on linear structures such as intrahepatic vessels. Then the operators drew a spherical marker along the target HCC nodule contour on the pretreatment US-MPR images so that the automatically synchronized spherical marker represented the original HCC contour on the post-treatment real-time US images. After completion of image synchronization, CEUS with perflubutane (Sonazoid) and RVS was performed. The acoustic power of the US machine was set at a mechanical index of 0.2. After bolus injection of perflubutane (Sonazoid) into an antecubital vein at a dose of 0.7 ml, the operators scanned regions covering the original target HCC nodules and surrounding hepatic parenchyma for up to 30 s, during which the patient kept holding his/her breath. Simultaneously, motion images were recorded. Injection of the contrast agent and scans were repeated as necessary.

### Assessments of RFA effects

Assessment of RFA effects was performed in real time and during frame-by-frame playbacks of the recorded motion images. Ablation was considered sufficient when an avascular area with a margin of several millimeters surrounded the spherical marker in all directions on the CEUS images.^15^ When HCC nodules were adjacent to vessels or near the liver surface, treatment was considered complete even if a margin was not obtained. When the US findings did not meet these criteria, additional RFAs were performed and CEUS and RVS with 3DUS data were used to assess the therapeutic effects again. Around the time that the CEUS and RVS using 3DUS data were performed, contrast-enhanced CT (CECT) or contrast-enhanced MR (CEMR) imaging was also performed, and their therapeutic effects were assessed by experienced radiologists in a blinded manner. The agreement rate for the therapeutic assessments between CEUS and RVS using 3DUS data and CECT or CEMR was calculated.

## Results

**FIGURE 1.**
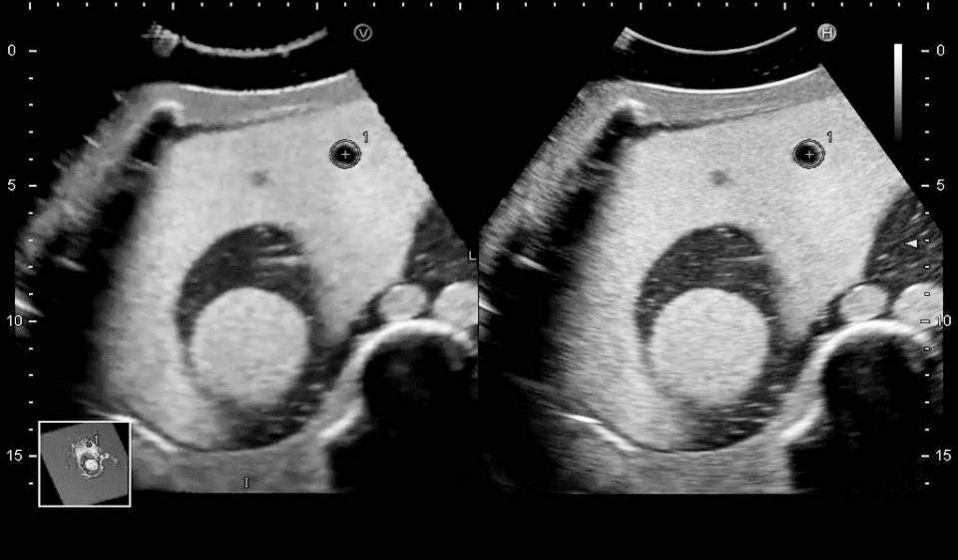
RVS synchronization using 3DUS data from a phantom model. US-MPR images from the 3D data (left) and real-time US images (right) are synchronized on the same US monitor. Furthermore, synchronized spherical markers (+, the center of the spherical marker) are displayed along the contour of a spherical structure of the phantom model on synchronized US-MPR and real-time US images. RVS = real-time virtual sonography; 3DUS = three-dimensional ultrasonography; MPR = multiplanar reconstruction

In every *in vitro* experiment, the US-MPR images from the 3D data and real-time US images were synchronized on the same US monitor (Figure 1). Furthermore, synchronized spherical markers were displayed along the contour of a spherical structure of the phantom model on the synchronized US-MPR and real-time US images.

**FIGURE 2.**
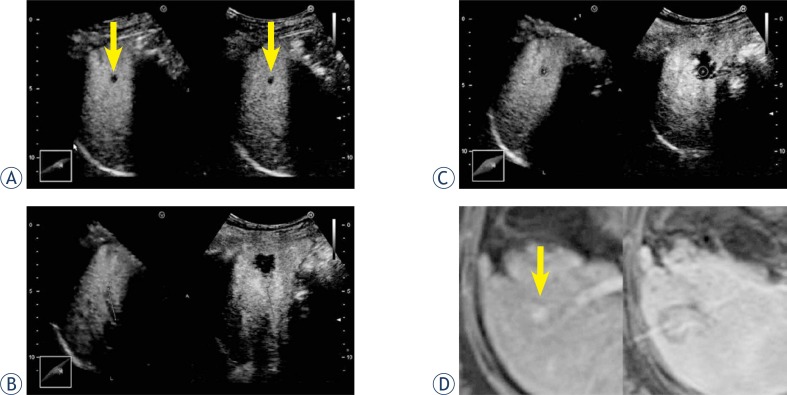
Assessment of the effects of RFA on HCC by combination of CEUS and RVS using 3DUS data. A 57-year-old female patient had a 7-mm HCC in segment 5. Sufficient ablation was achieved in a single session of RFA. **A.** CEUS and RVS using 3DUS data before RFA. The Kupffer phase data set was selected for RVS. The HCC nodule is shown as a perfusion defect (arrow). The US-MPR and real-time CEUS images are synchronized. **B.** CEUS and RVS using 3DUS data after RFA. The US-MPR (left) and real-time CEUS (right) images are adjusted with synchronized straight markers. Each marker is drawn on the same anterior branch of the right branch of the portal vein. **C.** CEUS and RVS using 3DUS data after RFA. Automatically synchronized spherical markers represent the HCC contour. An avascular area surrounds the sphere marker (+, the center of the spherical marker) with a margin of several millimeters on CEUS images, suggesting sufficient ablation. **D.** Dynamic MR imaging. Before RFA (left), the HCC nodule is shown as a hypervascular lesion (arrow). MR image obtained after RFA (right) shows a sufficient ablation zone. CEUS = contrast-enhanced ultrasonography; HCC = hepatocellular carcinoma; MR = magnetic resonance MPR = multiplanar reconstruction; RFA = radiofrequency ablation; RVS = real-time virtual sonography; 3DUS = three-dimensional ultrasonography

**FIGURE 3.**
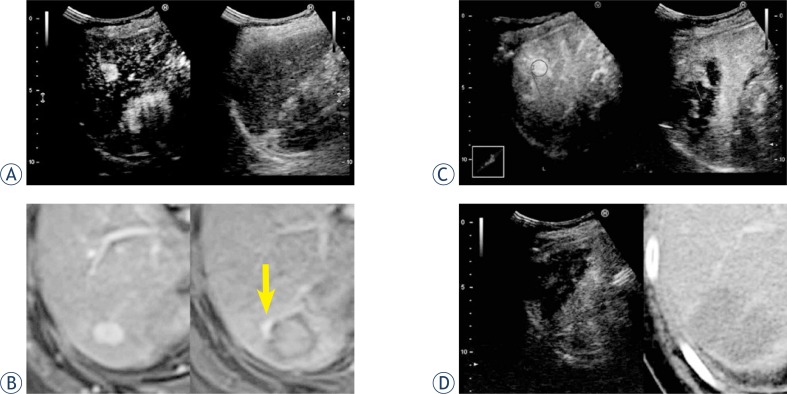
Usefulness of CEUS and RVS using 3DUS data in identifying HCC nodules requiring additional RFA. A 66-year-old female patient had a 13-mm HCC in segment 6. Because HCC was poorly visualized with conventional US, RFA was performed under CEUS guidance. After the first RFA session, dynamic MR showed a residual viable portion, but the second RFA session failed to ablate that portion. CEUS and RVS using 3DUS data clearly showed the portion requiring additional RFA. Therefore, a third RFA session was performed, which ablated the portion. **A.** CEUS before RFA. **B.** Dynamic MR imaging before RFA (left) and after the first RFA session (right). A residual viable portion is shown (arrow). **C.** CEUS and RVS using 3DUS data displays the portion requiring additional RFA (spherical marker). Each synchronized straight marker is drawn on the same posterior branch of the right branch of the portal vein. **D.** CEUS (left) and dynamic CT (right) suggest sufficient ablation. CEUS = contrast-enhanced ultrasonography; CT = computed tomography; HCC = hepatocellular carcinoma; MR = magnetic resonance; RFA = radiofrequency ablation; RVS = real-time virtual sonography; 3DUS, three-dimensional ultrasonography

Patient profiles are shown in Table 1. All patients were safely treated with RFA. The time required for RVS synchronization using stored 3D data ranged approximately from 10 to 15 min. The time tended to be prolonged in patients who could not continuously holding their breath. During CEUS and RVS, it was possible to assess whether an avascular area surrounded a spherical marker representing an original HCC contour. Furthermore, frame-by-frame playbacks of the recorded motion images permitted detailed reviews of the images, including measurement of ablated margins (Figure 2). Of the 13 patients, 2 underwent multiple sessions of RFA. In those patients, HCC-nodule portions requiring additional RFA were easily identified on US images (Figure 3). Additional sessions of RFA resulted in sufficient ablation for the patients. The total time required for RVS synchronization, CEUS, and RVS, including review of the recorded motion images, ranged approximately from 20 to 30 min. The agreement rate for the therapeutic assessments between CEUS and RVS using 3DUS data and CECT or CEMR was 100% in our series.

## Discussion

CECT and CEMR are standard methods for assessing the effects of RFA on HCC. By contrast, CEUS is currently employed as an adjunct method, although several studies have suggested that the usefulness of CEUS is comparable to that of the standard methods.^16^, ^17^ In the present study, we intended to actively use CEUS by combining RVS in the therapeutic assessment. On the basis of the feasibility, accuracy, and reproducibility of RVS synchronization using 3DUS data in *in vitro* experiments, we confirmed the applicability of CEUS and RVS using 3DUS data to the assessment of the effects of RFA on HCC. Our results showed that this method was feasible and its usefulness was comparable to that of CECT or CEMR. Furthermore, we noted that the present method has advantages over the standard methods. First, if post-treatment CECT or CEMR images suggest insufficient ablation, HCC-nodule portions requiring additional RFA need to be identified by US. Our method uses a single modality to enable both RFA and assessment of its effects. Furthermore, conventional side-by-side comparisons between pre- and post-treatment CECT or CEMR images often make accurate assessment of ablated margins difficult, especially when an ablated area is slightly larger than the original target HCC nodule. Our method uses a marking function that allows direct comparison of an avascular area formed by RFA with a spherical marker representing the original target nodule contour on real-time CEUS images.

The present method is unique in that it provides positional information about original HCC nodules on post-treatment real-time US images. Some earlier studies have shown the usefulness of synchronized MPR images in CEUS-based assessment of the effects of RFA on HCC.^18^,^19^ In those studies, CT-MPR images were used as reference images that were synchronized with CEUS images. The positions of target HCC nodules were identified by measuring the distance from the edge of the nodules to the architecture such as the surrounding organs. The researchers highlighted the usefulness of their method in decreasing the number of CECT images required for the therapeutic assessment. Our method is more advanced and convenient; therefore, it can be incorporated into the routine assessment of RFA effects, even though CECT and CEMR are currently the main assessment methods.

Patients who are allergic to contrast agents used during CT and MR imaging or those suffering from renal insufficiency are unsuitable for therapeutic assessments using CECT or CEMR. For these patients, other CE imaging methods are required. Perflubutane (Sonazoid) is less allergenic, and it can be used regardless of the patient’s renal function because it is excreted during exhalation.^20^ Therefore, our CEUS-based method is particularly useful for such patients.

Hiraoka *et al*. described the usefulness of RVS using 3DUS data in predicting the effects of RFA on HCC.^21^ The RVS system runs on a workstation connected to a US machine (EUB7500; Hitachi Medical Corporation). In their study, they used the system’s marking function to compare pretreatment US-MPR images with real-time US images 5 min after RFA. Because the extent of hyperechoic microbubbles induced by RFA is mostly proportional to the degree of ablation, they predicted that a sufficient margin was obtained when the microbubbles completely surrounded the spherical marker representing the original HCC nodule contour. The RVS system resulted in sufficient ablation and decreased the number of RFA sessions. The therapeutic effects were assessed by conventional CECT in the study conducted by Hiraoka *et al*. In contrast, we aimed to establish a US-based method to assess the effects of RFA. However, it is worth testing to see whether assessment of the extent of microbubbles immediately after RFA by our method is useful in predicting therapeutic effects.

The present method had some limitations. First, US image synchronization required the patients to hold their breath continuously, which was a difficult task for some patients. Second, our method may not be applied to HCC nodules located near US beam obstacles such as pulmonary air and calcium deposits. Third, the spherical marker contour did not completely overlap HCC nodules because their shapes were not perfect spheres. In cases with irregularly shaped HCC nodules, spherical marker contours should overlap HCC nodule portions that possibly have insufficient ablation margins.

This study suggested that the combination of CEUS and RVS using 3DUS data is feasible and useful for assessing the effects of RFA on HCC. The method uses advanced US technologies, and although its accuracy and reproducibility should be verified in a blinded study with a larger number of patients, we believe that the method will become an important modality and provide even greater benefits of US than those demonstrated previously for assessing the effects of RFA on HCC.

## Figures and Tables

**TABLE 1.** Patient profiles

**Parameter**	
Age (years)	70 ± 11^a^
Sex, Male/Female	5/8
Etiology of liver disease, HBV/HCV/Others	2/10/1
Child–Pugh grade, A/B	6/7
Location of HCC, Segment 3/4/5/6/7/8	1/2/2/3/2/4
Naive HCC nodule/Local recurrence nodule/Distant recurrence nodule	5/4/5
Maximum diameter (mm)	17 ± 6^a^
Electrode-tip exposure, 2 cm/3 cm	5/9
Number of electrode insertions, 1/2/3	8/4/2
Number of RFA sessions, 1/2/3	12/1/1

a Data are expressed as mean ± standard deviation

HBV = hepatitis B virus; HCC = hepatocellular carcinoma; HCV = hepatitis C virus; RFA = radiofrequency ablation
